# Finding good alternatives to hospitalisation: a data register study in five municipal acute wards in Norway

**DOI:** 10.1186/s12913-022-08066-3

**Published:** 2022-05-30

**Authors:** Vivian Nystrøm, Hilde Lurås, Tron Moger, Ann-Chatrin Linqvist Leonardsen

**Affiliations:** 1 Department of Health, Welfare and Organisation, Østfold University College, Postal Box Code (PB) 700, 1757 Halden, Norway; 2 Department of Health Management and Health Economics, University of Oslo, 1089 Blindern, Postal Box Code (PB), 0317 Oslo, Norway; 3 Health Services Research Unit, Akershus University Hospital, Postal box code (PB) 1000 1478 Lørenskog, Norway; 4 Institute of Clinical Medicine, Campus Ahus, University of Oslo, Nordbyhagen, Norway; 5 Østfold Hospital Trust, Grålum, Norway

**Keywords:** Health services research, Primary healthcare, Quality improvement, Register data, Regression analysis, Municipal acute wards, Pathways

## Abstract

**Background:**

In Norway, municipal acute wards (MAWs) have been implemented in primary healthcare since 2012. The MAWs were intended to offer decentralised acute medical care 24/7 for patients who otherwise would be admitted to hospital. The aim of this study was to assess whether the MAW represents the alternative to hospitalisation as intended, through 1) describing the characteristics of patients intended as candidates for MAWs by primary care physicians, 2) exploring the need for extended diagnostics prior to admission in MAWs, and 3) exploring factors associated with patients being transferred from the MAWs to hospital.

**Methods:**

The study was based on register data from five MAWs in Norway in the period 2014–2020.

**Results:**

In total, 16 786 admissions were included. The median age of the patients was 78 years, 60% were women, and the median length of stay was three days. Receiving oral medication (OR 1.23, 95% CI 1.09–1.40), and the MAW being located nearby the hospital (OR 2.29, 95% CI 1.92–2.72) were factors associated with patients admitted to MAW after extended diagnostics. Patients needing advanced treatment, such as oxygen therapy (OR 2.13, 95% CI 1.81–2.51), intravenous medication (OR 1.60, 95% CI 1.45–1.81), intravenous fluid therapy (OR 1.32, 95% CI 1.19–1.47) and MAWs with long travel distance from the MAW to the hospital (OR 1.46, 95% CI 1.22–1.74) had an increased odds for being transferred to hospital.

**Conclusions:**

Our findings indicate that MAWs do not represent the alternative to hospitalisation as intended. The results show that patients receiving extended diagnostics before admission to MAW got basic treatment, while patients in need of advanced medical treatment were transferred to hospital from a MAW. This indicates that there is still a potential to develop MAWs in order to fulfil the intended health service level.

**Supplementary Information:**

The online version contains supplementary material available at 10.1186/s12913-022-08066-3.

## Introduction

Many Western countries organise their health care systems within two governmental levels. Hospitals provide specialised medical services, while primary healthcare provides basic medical treatment and care [1–3]. In recent years, there has been a change towards decentralising medical treatment from hospitals to primary health care, and different initiatives have been implemented and tested [4–6].

In Norway, municipal acute wards (MAWs) were introduced in 2012 as an alternative to hospitalisation for patients with a clarified diagnosis who need acute medical treatment, but who are not in need of specialist health care services [7, 8]. Patients admitted to a MAW must be over 18 years old and have an acute deterioration of an already known condition and/or have a clarified condition that is expected to be fully treated within approximately three days [7]. National guidelines for selecting patients suitable for MAW admission are broad, and great emphasis has been placed on local self-government in selecting patients [7–9]. However, the patients must be assessed by either a general practitioner, an out-of-hours physician or a nursing home physician, all of whom are primary care physicians (PCPs), before MAW admission. More extended diagnostics beyond what the primary care physicians can offer, such as x-ray, ultrasound images or blood samples, can be provided in the hospital before admission to the MAW. Based on such extended diagnostics hospital physicians may claim a need for hospitalisation for the patient instead of an admission to a MAW.

Patients admitted to a MAW may experience a deterioration beyond what is thought appropriate for the MAW to handle, and are consequently transferred to hospital [10]. The selection of patients suitable for MAW admission thus can be challenging, and studies indicate that treating patients outside hospitals causes uncertainty for the responsible healthcare personnel [11–14].

The structure, equipment and range of services offered in MAWs varies. Some MAWs are organised as inter-municipal units, some are located in relation to a hospital, others close to a casualty clinic or a nursing home. Some MAWs have physicians and nurses present 24 h, while others have to use PCPs from a casualty or a nursing home for consultations. The number of beds in a MAW varies from small units with 3 beds or less to large units with 15 beds or more [15]. There is no national guidelines regarding medical-technical equipment and diagnostics that should be available or present at a MAW; i.e. some MAWs offer advanced diagnostics such as x-ray or computer tomography, while others do not have these possibilities [16, 17].

Whether the MAWs represent the alternative to hospitalisation that was intended from the health authorities is so far inconclusive. For example, one study showed that 52.7% of MAW patients admitted from home were discharged to nursing homes after a stay, indicating that MAWs were used as a pathway for such admission [18]. Another study argued that MAW patients were very old and had complex health problems when admitted, resulting in a prolonged length of stay and indicating that the patients’ needed comprehensive care rather than specialised medical treatment [19].

This study aimed to assess whether the MAW represents the alternative to hospitalisation as intended. Our objectives were to 1) describe the characteristics of patients intended as candidate for MAWs by primary care physicians, 2) explore the need for extended diagnostics prior to admission in MAWs, and 3) explore factors associated with patients being transferred from the MAWs to hospital.

## Materials and methods

The study adheres to the Reporting of Studies Conducted using Observational Routinely Collected Data (RECORD) guidelines [20]. All methods were carried out in accordance with relevant guidelines and regulations (see ethics approval).

### Study design and data sources

The study had a prospective, observational design, based on anonymous data collected from five MAWs in southeastern Norway in the period 2014–2020. At discharge, nurses in administrative positions at the MAWs complete a mandatory reporting form with anonymised patient information.

The mandatory forms contain detailed characteristics of the MAW admissions: ‘patients’ gender’, ‘patients’ age’, ‘treatment provided’, and ‘the International Classification in Primary Care (ICPC-2) main diagnosis leading to the admission’. Information about ‘ICPC-2 additional diagnosis 1 at admission’, and ‘ICPC-2 additional diagnosis 2 at admission’ are based on patients’ comorbid conditions, but are not the reason for admittance to the MAW. The forms also contain information about the date of admission, whether ‘the admission was day/evening/night’, whether ‘the admission was weekend/weekday’, ‘where the patient is admitted from’, ‘who the referring primary care physician is’, ‘date of discharge’ and ‘where the patient is discharged to’. The information collected in the forms are registered in a data file in each MAW. The files from the five MAWs are then merged into one file in the analysis department at the hospital.

The study was conducted within one hospitals’ catchment area in South-eastern Norway. The five MAWs in this region were established in the period 2012–13. They were organised as inter-municipal units covering an area of 12 municipalities, with approximately 320 000 inhabitants. Table 1 gives and overview of the five MAWs’ characteristics.

**Table 1:** Characteristics of the five MAWs

	**MAW 1**	**MAW 2**	**MAW 3**	**MAW 4**	**MAW 5**
Number of beds	11	8	10	4	7
Travel distance to the hospital by car, minutes	30	15	30	45	45
Physician(s) present Weekdays (08–16)	yes	yes	yes	yes	yes
Physician(s) present Weekends (09–15)	yes	yes	yes	yes	no
Nurse(s) present	24/7	24/7	24/7	24/7	24/7
Travel distance to the casualty by car, minutes	0	0	5	5	15^a^
Co-located with short-term care	yes	yes	yes	yes	yes
X-ray available	daytime	daytime	daytime	-	mobile X-ray to days per week
Laboratory haemoglobin, WBC differential, CRP, glucose and urine examinations available	daytime	daytime	daytime	daytime	daytime
Blood gas available	at causality	at causality	yes	no	at causality
ECG available	yes	yes	yes	yes	yes
Bladder scanning available	yes	yes	yes	yes	yes

*Abbreviations*: *MAW* Municipality acute ward, *Casualty* after-hours emergency services provided by primary care physicians in dedicated locations, as consultation wards (no treatment), *WBC* differential white blood cell differential, *CRP* C-reactive protein, *ECG* Electrocardiogram, *Blood gas* a group of tests that are performed together to measure the pH and the amounts *PaO2* and *PaCO2* (arterial pressure of oxygen and carbon dioxide), bicarbonate (*HCO3*), lactate, Haemoglobin (*Hb*), electrolytes, and blood sugar present in a sample of blood, *Bladder scanning* ultrasonic reflections measures the amount of urine inside the bladder

^a^ means that travel time by car to the casualty was 15 min in 2019 and 2020. Travel distance by car in 2014 – 2019 was 0 min

**Fig. 1:**
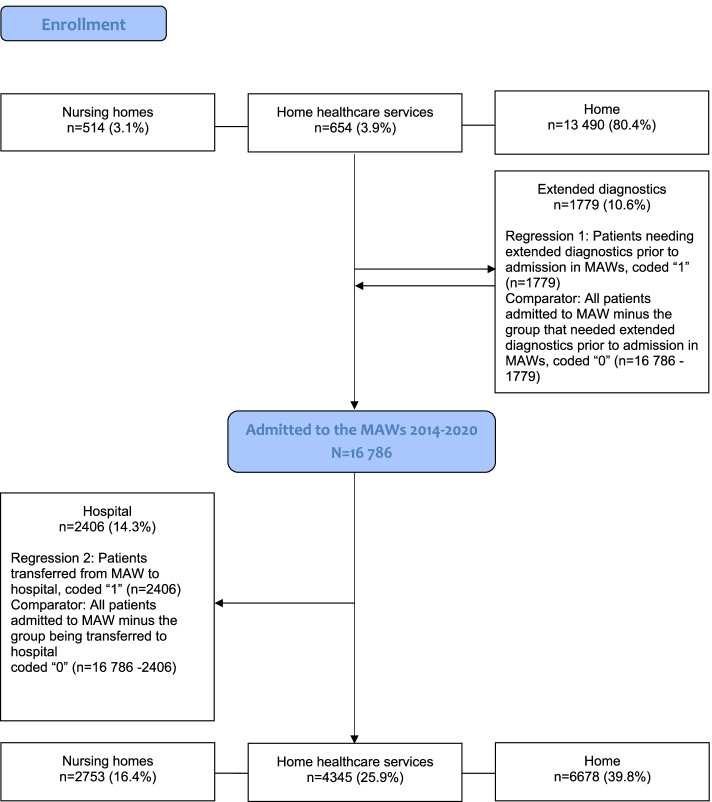
Patients admitted to the MAWs in the period 2014–2020. Regression 1 is presented in Table 3, while regression 2 is presented in Table 4

All adults ≥ 18 years admitted to one of the five MAWs during the study period were included (see Fig. 1).

### Variables collected

#### Outcome variables

The outcome variables of this study were 1) patients intended for MAW needing extended diagnostics, and 2) patients being transferred from the MAW to the hospital. The variable ‘needing extended diagnostics’ was coded yes/no. The variable ‘transferred to hospital’ was coded yes/no and was constructed based on the original variable in the registry ‘where the patient is discharged to’. This means that there is some overlap between the two outcomes, because some of the patients needing extended diagnostics also were transferred from the MAW to hospital.

#### Treatment

Each patient can be registered with several medical treatment variables. Treatment variables are ‘Oral medication’, ‘Intravenous fluid therapy’, ‘Intravenous medication’, ‘Mobilisation and pain relief’, ‘Nebuliser therapy’, ‘Oxygen therapy’, ‘Observation’, ‘Emptying regime/constipation’, ‘Bladder catheterisation’, ‘Wound therapy’, ‘Blood transfusion’, ‘Nutritional therapy’, ‘Physical therapy’ (see Additional file 2). The variables were coded yes/no, based on treatment received.

#### Diagnosis

The patients’ diagnosis are coded according to the International Classification of Primary Care (ICPC-2) [21]. The variable ‘ICPC-2 main group’ includes 17 alternatives relating to symptoms from different organ systems ‘Respiratory’, ‘Musculoskeletal’, ‘Digestive’, ‘Urological’, ‘Endocrine/metabolic and nutritional’, ‘General and unspecified’, ‘Psychological’, ‘Cardiovascular’, ‘Blood, blood forming organs and immune mechanism’, ‘Neurological’, ‘Skin’, ‘Pregnancy, childbearing, family planning’, ‘Female genital’, ‘Male genital’, ‘Social Problems’, ‘Ear’, ‘Eye’ (see additional file 2). Each patient is registered with one main diagnosis, and other registered diagnoses are included as comorbidities. The variables were coded yes/no.

#### Comorbidities

‘Number of registered comorbidities’ was calculated from the registered variables ‘alternative ICPC-2 code 1 at admission’ and ‘alternative ICPC-2 code 2 at admission’. They were selected according to the literature and were assessed by an experienced chief physician and a specialist nurse [10, 22, 23] (see Additional file 1).

#### MAW admission and discharge

‘Where the patient is admitted from’ is categorised as ‘from home’, ‘from home healthcare services’, or ‘from nursing home’. ‘Where the patient is discharged to’ is categorised as ‘to home’, ‘to home healthcare services, ‘to short-term care nursing home’ or ‘to hospital’. ‘Who the referring primary care physician is’ is categorised as ‘general practitioner’, ‘out-of-hour physician’ or ‘nursing home physician’. Admitting time is categorised as ‘day’, ‘evening’ and ‘night’. ‘Length of stay’ was calculated as ‘date of discharge’ minus ‘date of admission’.

#### Cleaning methods

Plotting errors were removed and coded as ‘system missing’. Age values outside the range of the MAW admission guidelines were removed and coded as ‘system missing’. In the analyses, we implicitly assumed that the values were missing at random, with a missing range from 1–913 on individual variables. All the variables were discussed for content both with managers in the MAWs who manually did the plotting, with physicians working in the MAWs, with statisticians at the analysis department at the Hospital Trust, and between the authors.

### Analysis

Descriptive statistics are presented as numbers and percentages and as medians, means and standard deviations (SDs), as appropriate. To obtain associations between outcome variables and predictors/covariates, we first conducted univariate logistic regressions per outcome variable, i.e., 1) patients needing extended diagnostics prior to MAW admission, and 2) patients being transferred from the MAW to the hospital. Second, we conducted one multiple logistic regression analysis per outcome variable to obtain odds ratios (ORs) and 95% confidence intervals (CIs). Statistical significance was set at *p* < 0.05.

Due to missing values for individual variables, there is a slight variation in the numbers included in the analyses (see Table 2). We were also unable to estimate effects in some of the ICPC-2 main groups and some of the treatment options in the multiple analysis due to insufficient observations (see Tables 3 and 4). Therefore, these variable categories were removed from the logistic regression analysis. The removed treatment variables were ‘wound therapy’, ‘blood transfusion’, ‘nutritional therapy’ and ‘physical therapy’. The ICPC-2 groups removed were ‘blood, blood forming organs and immune mechanism’, ‘pregnancy, childbearing, family planning’, ‘female genital’, ‘male genital’, ‘social problems’, ‘ear’ and ‘eye’. All analyses were performed with IBM Statistical Package for the Social Sciences (SPSS) Statistics version 27 [24].

## Results

### Descriptives of patients intended as candidate for MAWs by primary care physicians

**Table 2:** Descriptive statistics for patients admitted to MAW in the period 2014 to 2020, *N* = 16 786

	**Patients intended for MAW admission, *N* = 16786**n (%)		**Extended diagnostics, *n* = 1779** n (%)		**Transfer to hospital, *n* = 2406**n (%)	
**Treatment** (missing:913)						
Oral medication	9682 (57.7)		1146 (64.4)		1306 (54.3)	
Intravenous fluid therapy	5482 (32.7)		1275 (71.7)		943 (39.2)	
Intravenous medication	4658 (27.7)		481 (27.0)		868 (36.2)	
Mobilisation and pain relief	3755 (22.4)		482 (27.0)		439 (18.2)	
Observation	2949 (17.6)		367 (20.6)		461 (18.7)	
Oxygen therapy	1763 (10.5)		196 (11.1)		404 (16.8)	
Nebuliser therapy	1904 (11.4)		201 (11.3)		301 (12.5)	
Emptying regime/constipation	790 (4.7)		69 (3.9)		98 (4.1)	
Bladder catheterisation	477 (2.8)		48 (2.7)		78 (3.2)	
Wound therapy	467 (2.8)		50 (2.8)		51 (2.1)	
Blood transfusion	433 (2.6)		10 (0.6)		11 (0.5)	
Nutritional therapy	355 (2.1)		32 (1.8)		58 (2.4)	
Physical therapy	199 (1.1)		31 (1.7)		14 (0.6)	
**ICPC-2 main groups** (missing:873)						
Respiratory	3814 (22.7)		492 (33.4)		595 (24.7)	
Musculoskeletal	2633 (15.7)		375 (21.1)		334 (13.9)	
Digestive	1619 (9.6)		114 (6.4)		300 (12.5)	
Urological	1504 (9.0)		157 (8.8)		253 (10.5)	
Endocrine/ metabolic and nutritional	1362 (8.1)		78 (4.4)		177 (7.4)	
General and unspecified	1318 (7.8)		111 (6.3)		233 (9.7)	
Psychological	978 (5.8)		64 (3.6)		113 (4.7)	
Cardiovascular	603 (3.6)		85 (4.8)		114 (4.7)	
Blood, blood forming organs and immune mechanism	604 (3.6)		12 (6.7)		31 (1.3)	
Neurological	585 (3.5)		75 (4.2)		69 (2.9)	
Skin	573 (3.4)		60 (3.4)		86 (3.6)	
Pregnancy, childbearing, family planning	163 (1.0)		36 (2.0)		17 (0.7)	
Female genital	41 (0.2)		1 (< 0.0)		11 (0.5)	
Male genital	38 (0.2)		0 (0.0)		8 (0.3)	
Social Problems	38 (0.2)		3 (< 0.0)		2 (0.1)	
Ear	33 (0.2)		2 (< 0.0)		1 (< 0.0)	
Eye	7 (< 0.1)		0 (0.0)		2 (0.1)	
**Comorbidities** (missing:0)						
No comorbidities	14,078 (83.9)		1447 (81.3)		2027 (84.2)	
One comorbidity	2212 (14.4)		298 (16.8)		338 (14.1)	
Two comorbidies	396 (1.8)		34 (8.6)		41 (1.7)	
**Gender** (missing:42)						
Female	10,051 (59.9)		1085 (61.0)		1354 (56.3)	
Male	6693 (39.9)		691 (38.8)		1049 (43.6)	
**Municipal acute Ward** *(missing:0)*						
MAW 1	4630(27.6)		463(26.0)		548(22.8)	
MAW 2	2111(12.6)		428(24.0)		296(12.3)	
MAW 3	4217(25.1)		392(22.0)		517(21.5)	
MAW 4	1823(10.9)		181(10.2)		308(12.8)	
MAW 5	4005(23.9)		315(17.7)		737(30.6)	
**Referred from** (missing:432)						
General Practitioner	6900 (41.1)		651 (36.6)		916 (38.1)	
Out-of-hour physician	9337 (55.6)		997 (56.0)		1430 (59.4)	
Nursing home physician	117 (0.7)		4 (0.0)		15 (0.6)	
**Admitting time** (missing:0)						
Day	5287 (31.5)		739 (41.5)		736 (30.6)	
Evening	8349 (49.7)		454 (25.5)		1222 (50.8)	
Night	3150 (18.8)		586 (32.9)		448 (18.6)	
**Admission Weekend/ Weekday** (missing:0)						
Weekend	3911 (23.3)		434 (24.4)		601 (25.0)	
Weekday	12,875 (76.7)		1345 (75.6)		1805 (75.0)	
**Admitted from** (missing:349)						
Home	13,490 (80.4)				2025 (84.2)	
Home healthcare services	654 (3.9)				97 (4.0)	
Nursing homes	514 (3.1)				83 (4.4)	
**Discharged to** (missing:604)						
Home	6678 (39.8)		693 (39.0)			
Home with home-nursing	4345 (25.9)		562 (31.6)			
Nursing home (short time care)	2657 (15.8)		287(16.1)			
Nursing home (long time care)	96 (0.6)		7 (0.4)			
Hospital	2406 (14.3)		160 (9.0)			
	**Total**		**Extended diagnostics**		**Hospital**	
	Mean/Median	SD/IQR	Mean/Median	SD/IQR	Mean/ Median	SD/IQR
**Age** *(missing:166)*	73.5/78	17.8/66–86	75/80	17.2/68–87	72.6/77	17.7/65–86
**Length of stay** *(missing:126)*	3.4/3	3.3/1–5	3.8/3	3.3/2–5	2.9/1	2.7/1–3

*N* Number, *MAW* Municipal acute Ward, *ICPC-2* The International Classification of Primary Care-2; Age in years; Length of stay in days, *SD* Standard deviation, *IQR* Interquartile range

The left column in Table 2 gives a descriptive summary of characteristics of patients intended as candidate for MAWs by primary care physicians from 2014 to 2020 (*n* = 16 786). Of these, 60% were female, the median age was 78 years with inter quartile (IQ) range from 66 to 86 years, and the median length of stay was three days with an IQ range from one to five days. The most frequent cause of admission was symptoms in the ICPC-2 main groups ‘respiration’ and ‘musculoskeletal’. Treatments most commonly provided in the MAWs were ‘oral medication’, followed by ‘intravenous fluid therapy’, ‘intravenous medication’, ‘mobilisation and pain relief’, ‘observation’, ‘nebuliser therapy’, and ‘oxygen therapy’. A majority of the patients (80.4%) were ‘admitted from home’. After a stay at a MAW, 41.7% were ‘discharged to home healthcare services’ or to ‘short-term care nursing home’. More patients were ‘admitted from an out-of -hours physician’ at the casuality than from ‘a general practitioner’ (55.6% versus 41.1%).

### Patients needing extended diagnostics prior to admission in MAWs

Table 2 (middle column) shows that 1 779 (10.6%) patients in the sample were assessed as needing extended diagnostics before admittance to a MAW. These patients were ‘older’, had more ‘comorbidities’ and had longer ‘length of stay’ compared to patients not needing extended diagnostics. Patients with diagnosis from ICPC-2 groups ‘respiratory’ and ‘musculoskeletal’ were most frequent (totally 38,4% versus 54.5%). Several were ‘admitted during night’ compared to the whole population (32.9% versus 18,8%). More patients in this group were ‘sent to home healthcare services’ than the other MAW patients (31.6% versus 25.9%). They also more frequently received treatment with ‘oral medication’ (64.4% versus 57.7%) than the group in total.

**Table 3:** Univariate and multiple logistic regressions on patients needing extended diagnostics (total number of patients included in the regression analysis, *n* = 13,987)

	**Univariate regression**	**Multiple regressions**
	OR (95% Cl)	OR (95% Cl)
**Treatment (reference no treatment on each treatment alternative)**		
Oral medication	1.36 (1.23–1.51)*	1.23 (1.09–1.40)*
Intravenous fluid therapy	0.81 (0.72–0.90)*	0.90 (0.73–0.95)*
Intravenous medication	0.97 (0.86–1.08)	1.02 (0.89–1.16)
Mobilization and pain relief	1.32 (1.18–1.47)*	1.05 (0.91–1.20)
Nebulizer therapy	0.99 (0.85–1.16)	0.73 (0.59–0.90)*
Oxygen therapy	1.06 (0.90–1.24)	1.08 (0.88–1.33)
Observation	1.25 (1.10–1.41)*	1.25 (1.08–1.45)*
**ICPC-2 main group (reference respiratory)**		
General and unspecified	0.63 (0.51–0.78)*	0.61 (0.48–0.77)*
Digestive	0.51 (0.41–0.63)*	0.53 (0.42–0.67)*
Cardiovascular	1.11 (0.87–1.42)	0.98 (0.73–1.30)
Musculoskeletal	1.11 (0.96–1.29)	1.04 (0.87–1.24)
Neurological	0.99 (0.76–1.28)	0.86 (0.64–1.16)
Psychological	0.47 (0.36–0.62)*	0.44 (0.33–0.60)*
Skin	0.79 (0.59–1.05)	0.81 (0.59–1.11)
Endocrine/metabolic and nutritional	0.42 (0.33–0.54)*	0.45 (0.34–0.59)*
Urological	0.79 (0.65–0.95)*	0.67 (0.54–0.83)*
**Comorbidities (reference comorbidity = 0)**		
One Comorbidity	1.21 (1.06–1.39)*	1.25 (1.08–1.46)*
Two Comorbidities	1.11 (0.78–1.60)	1.24 (0.84–1.85)
**Gender (reference female)**		
**Male**	0.95 (0.86–1.05)	1.03 (0.92–1.16)
**Age/10**	1.06 (1.02–1.09)*	1.09 (1.05–1.13)*
**Length of stay (in days)**	1.03 (1.02–1.05)*	1.01 (1.00–1.03)
**Municipal acute Ward (reference MAW 1)**		
MAW 2	2.28*(1.97–2.63)	2.29*(1.92–2.72)
MAW 3	0.92 (0.79–1.05)	1.00 (0.84–1.19)
MAW 4	1.11 (0.92–1.33)	1.24 (1.00–1.54)*
MAW 5	0.76 (0.66–0.89)*	0.94 (0.78–1.12)
**Referred from (reference general practitioner)**		
Out-of-hour physician	1.15 (1.03–1.27)*	0.44 (0.38–0.51)*
Nursing home physician	0.36 (0.13–0.99)*	0.41 (0.09–1.79)
**Admitting time (reference day)**		
Evening	0.36 (0.32–0.40)*	0.25 (0.21–0.29)*
Night	1.42 (1.26–1.60)*	1.83 (1.59–2.10)*
**Admission Weekend/Weekday (reference weekend)**		
Weekday	0.94 (0.84–1.05)	0.80 (0.69–0.93)*
**Discharged to (reference home)**		
Home healthcare services	1.28 (1.14–1.44)*	1.09 (0.94–1.26)
Hospital	0.62 (0.52–0.74)*	0.60 (0.49–0.73)*
Nursing home (short time care)	1.06 (0.91–1.22)	0.86 (0.72–1.03)
Nursing home (long time care)	0.70 (0.33–1.53)	1.28 (0.56–2.91)

Regressions were logistic

*OR* Odds ratio, *CI* Confidence interval, *MAW* Municipal acute ward, *ICPC-2* The International Classification of Primary Care-2, *N* Number in multiple analysis

^*^p-value significant at level 0.05

Table 3 shows the results of the logistic regression analysis on the effects of the explanatory variables of patients ‘needing extended diagnostics’ prior to MAW admission. In the univariate logistic regression analyses, receiving ‘oral medications’, ‘mobilisation and pain relief’ or ‘observation’ conferred a higher odds for needing extended diagnostics. Patients with symptoms in the ICPC-2 main group ‘general and unspecified’, ‘digestive’, ‘psychological’, ‘endocrine/metabolic/ nutritional’ and ‘urological’ had lower odds for needing extended diagnostics compared to the ICPC-2 main group ‘respiratory’. MAWs with the shortest ‘travel distance to the hospital’, patients ‘admitted from the causalty’ and ‘admittance at night’ were positively associated with ‘needing extended diagnostics’. ‘Needing extended diagnostics’ was positively associated with ‘discharge to home healthcare services’.

In the multiple regression model, ‘receiving oral medications’ and ‘observation’ were still associated with a higher likelihood of needing extended diagnostics prior to MAW admission. The ICPC-2 main group ‘general and unspecified’, ‘digestive’, ‘psychological’, ‘endocrine/metabolic/nutritional’ and ‘urological’ had in the multiple model lower odds for needing extended diagnostics compared to ICPC-2 main group ‘respiratory’. Further, the MAW with the shortest ‘travel distance to the hospital’ and ‘patients admitted at night’ had the highest odds of being needing extended diagnostics prior to MAW admission.

### Patients being transferred to hospital

Table 2 (right column) shows that 2 406 (14.3%) patients were ‘transferred to hospital’ from the MAWs. The median ‘length of stay’ was one day, and there were more ‘men’ (43.6% versus 39.9%) compared to the MAW group in total. More patients in this group were ‘referred from an out-of-hours physician’ (59.4% versus 55.6%). The most frequent diagnosis was from ICPC-2 main group ‘respiratory’. Patients from the ICPC-2 groups ‘digestive’ og ‘urological’ were more often transferred to hospital compared to other MAW patients. Furthermore, more patients received treatment with ‘intravenous medications’ (36.2% versus 27.7%), as well as ‘oxygen therapy’ (16.8% versus 10.5%).

Table 4 shows the results of the logistic regression analysis on the effects of the explanatory variables on the likelihood that the patient was transferred to hospital. The univariate-variable model showed that receiving ‘intravenous fluid therapy’, ‘intravenous medications’, or ‘oxygen therapy’ were highly associated with transfer to hospital, as were symptoms in the ICPC-2 main groups ‘digestive’ and ‘cardiovascular, as compared to ‘respiratory’. The longest travel distance from the MAW to the hospital, ‘referred from out-of-hour physician’ from the casualty and ‘male’ gender were also positively associated with transfer to hospital. Patient ‘being admitted to MAW after extended diagnostics’ had lower odds for being ‘transferred to hospital’.

**Table 4:** Univariate and multiple logistic regressions on patients transferred from MAW to hospital during the stay at MAW (total number patients included in the regression analysis, *n* = 14 202)

	**Univariate regressions**	**Multiple regression**
	**OR (95% Cl)**	**OR (95% Cl)**
**Treatment (reference no treatment)**		
Oral medication	0.85 (0.78–0.93)*	0.86 (0.77–0.95)*
Intravenous fluid therapy	1.40 (1.28–1.53)*	1.32 (1.19–1.47)*
Intravenous medication	1.58 (1.44–1.73)*	1.60 (1.45–1.81)*
Mobilization and pain relief	0.75 (0.67–0.83)*	0.95 (0.65–1.08)
Nebulizer therapy	1.14 (1.00–1.30)	0.78 (0.65–0.93)*
Oxygen therapy	1.93 (1.71–2.18)*	2.13 (1.81–2.51)*
Observation	1.13 (1.02–1.27)*	1.29 (1.13–1.48)*
Emptying regime/constipation	0.84 (0.68–1.04)	0.88 (0.69–1.13)
Bladder catheterization	1.17 (0.92–1.50)	1.11 (0.84–1.46)
Wound therapy	0.73 (0.54–0.98)*	0.82 (0.60–1.14)
Nutritional therapy	1.27 (0.88–1.56)	1.55 (1.14–2.12)*
**ICPC2 main group (reference respiratory)**		
General and unspecified	1.16 (0.98–1.37)	1.07 (0.89–1.29)
Digestive	1.23 (1.06–1.43)*	1.13 (0.94–1.35)
Cardiovascular	1.26 (1.01–1.57)*	1.31 (1.02–1.68)*
Musculoskeletal	0.79 (0.68–0.91)*	1.04 (0.87–1.24)
Neurological	0.72 (0.55–0.94)*	0.69 (0.52–0.92)*
Psychological	0.71 (0.57–0.88)*	0.67 (0.52–0.86)*
Skin	0.96 (0.75–1.22)	1.12 (0.86–1.47)
Endocrine/metabolic/nutritional	0.81 (0.68–0.97)*	0.75 (0.61–0.92)
Urological	1.09 (0.93–1.29)	1.18 (0.99–1.42)
**Comorbidities (reference comorbidities = 0)**		
One comorbidity	0.97 (0.86–1.10)	1.06 (0.93–1.22)
Two comorbidities	0.96 (0.69–1.33)	1.05 (0.73–1.51)
**Gender (reference female)**		
Male	1.19 (1.09–1.30)*	1.16 (1.05–1.27)*
**Age/10**	0.97 (0.96–0.99)*	1.05 (1.02–1.08)*
**Municipal acute Ward (reference MAW 1)**		
MAW 2	1.22 (1.04–1.41)*	0.92 (0.77–1.10)
MAW 3	1.04 (0.92–1.18)	0.83 (0.72–0.96)*
MAW 4	1.51 (1.30–1.76)*	1.46 (1.22–1.74)*
MAW 5	1.68 (1.49–1.89)*	1.04 (0.91–1.20)
**Referred from (reference general practitioner)**		
Out-of-hour physician	1.18 (1.08–1.29)*	1.06 (0.93–1.21)
Nursing home physician	0.96 (0.56–1.66)	1.13 (0.51–2.50)
**Admitting time (reference day)**		
Evening	1.06 (0.96–1.17)	1.03 (0.91–1.17)
Night	1.02 (0.90–1.16)	0.85 (0.74–0.98)*
**Admission Weekend/Weekday (reference weekend)**		
Weekday	0.90 (0.81–0.99)*	1.01 (0.89–1.14)
**Admitted from (reference home)**		
Home healthcare services	0.99 (0.80–1.23)	1.11 (0.87–1.43)
Extended diagnostic	0.56 (0.47–0.66)*	0.63 (0.52–0.76)*
Nursing homes	1.09 (0.86–1.39)	0.86 (0.64–1.15)

Regressions were logistic

*OR* Odds ratio, *CI* Confidence interval, *MAW* Municipal acute ward, *ICPC-2* The International Classification of Primary Care-2, *N* Number in multiple analysis

^*^ *p*-value significant at level 0.05

In the multiple regression model, the variables describing advanced medical treatment (i.e., ‘intravenous fluid therapy’, ‘intravenous medications’, and ‘oxygen therapy’) had higher odds of transfer to hospital. Further, patients treated in the MAW with the longest travel distance had the highest odds of being transferred to hospital. The regression model showed still higher odds for transfer to hospital if the patient was ‘male’. Patient ‘send for extended diagnostics’ had still lower odds for being ‘transferred from MAW to hospital.

## Discussion

The aim of this prospective observational study was to assess whether the MAW represents the alternative to hospitalisation as intended by policymakers. Our results show that patients intended as candidates for MAWs by primary care physicians received basic medical treatment such as oral medication. Many patients also needed extended diagnostics in hospital before being admitted to a MAW. Patients who were transferred to hospital during the stay at a MAW were in need of advanced medical treatment, such as intravenous fluid therapy, intravenous medication and oxygen therapy.

Our findings show that patients treated at MAWs mostly receive basic rather than specialised medical treatment. Thus, the MAW appears to represent an intermediate unit rather than an alternative to the hospital. This is supported by studies claiming that the MAW represents an additional health service to already existing services [12, 14, 19]. Originally, the intention of the MAW was to establish an alternative to hospitalisation, particularly suitable for patients with a clarified condition or an acute deterioration of an already known condition. [7, 8]. Implementation of the MAWs has contributed to a reduction in acute medical admissions and has led to a 1.9% reduction in hospitalisations for patients aged over 80 years [18, 25, 26], which could indicate that the MAWs do replace hospitalisations. However, our findings show that the medical treatment provided at the MAW is rather basic and hence could alternatively have been managed at home with the help of home healthcare services. This indicates that the home healthcare services capacity or competence might be too low. Hence, capacity building in home healthcare services might further reduce the pressure on hospitals. Our findings do not necessarily indicate that there has been an improper or wrong use of MAWs; rather, the MAW fills a healthcare service gap in the interface between hospitals and homes.

Moreover, our results show that patients admitted to the smallest MAW who also had the longest travel distance by car to the hospital were most likely to be transferred to hospital. In contrast, patients admitted to one of the biggest MAWs with shorter travel distance by care to hospital had lower odds for being transferred to the hospital. This may indicate that the healthcare personnel are more uncomfortable managing the risks of treating acutely ill patients when they are farther from the hospital [27]. In addition, our findings may indicate that the size of the MAW has an impact on how severe conditions the personnel can handle. A national study shows that MAWs located inside nursing homes had significantly more shifts with only one Registered Nurse (RN) on duty compared to MAWs located separately from other health care services [28]. Studies also indicate that there is a wide variation in whether patients are transferred to hospital from the MAW for further medical treatment, ranging from 7.8% to 23.6% [10, 18].

The extended diagnostics in hospital was used for one out of ten patients. The extended diagnostics service has become a better-known opportunity for clinicians to ensure that patients admitted to the MAW receive the right diagnosis; that is, the use of X-rays, laboratory tests and specialist assessment by hospital physicians are assumed to give the patients a more clarified diagnosis [7, 12]. However, most patients in MAWs are old, with a high degree of frailty [12, 19]. The risks of transporting frail elderly individuals are diverse, and their transport is associated with a significantly increased risk of morbidity and mortality [29–31]. Therefore, the decision to transport patients to hospital for extended diagnostics before admittance to MAW should be based on a weighing of the necessity of being diagnosed in hospital against the potential risk involved. A frailty index could help to identify older people at risk of health decline and mortality, guiding clinicians in their decision-making [32].

There has been a scepticism about the lack of physician coverage at MAWs throughout the day [12, 33]. It may be argued that for the MAW to be an acceptable alternative to hospitals, their equipment and expertise must be similar to those of hospitals. However, there are several studies that indicate that there is no threat to patient safety to be treated in nurse-led units [34, 35], and two single-centre randomised controlled studies found reduced morbidity after treatment in this kind of decentralised healthcare service [36, 37]. In a prospective observational study, it was shown that a ‘triage early warning score (TEWS)” above 2 indicates that patients have critical symptoms, need advanced treatment, and are more likely to be transferred to hospital from a MAW [10]. Hence, implementing the use of the TEWS score at diagnosis may guide clinicians in deciding which patients are suitable for admission to a MAW and which patients should be admitted to hospital.

The success of MAWs in Norway is that the patients themselves want to go there, but there are concerns regarding patient safety from the view of PCPs [12, 38, 39]*.* The selection of patients suitable for admittance to such healthcare services as the MAW is still considered a challenge [12, 40]. Studies indicate the potential to use machine algorithms to ensure that the right patients are directed to the right service level [40]. In addition, telemedicine has been suggested as well suited to guide medical decisions in more rural areas [41]. Our findings indicate that such solutions could be beneficial to help physicians refer patients to the right healthcare service and level.

### Strengths and limitations

One strength of this study is that it is based on a large and complete dataset covering five MAWs over a seven-year period, which allowed more reliable estimates than previous studies in the field. The five MAWs differed in size, geographical location, staffing and diagnostic opportunities and were also similar to community-based units internationally. This strengthens the external validity and generalisability of our findings.

The analyses presented here are explorative, and the significant findings should ideally be replicated in further studies. A limitation in this study is that we did not had data on patients needing extended diagnostics as assessed by PCPs who were hospitalised, but only on patients that hospital physicians agreed were suitable for treatment in a MAW and consequently were admitted as intended. This may bias the findings. This group can be healthier than those who were hospitalised. Hence, factors such as distance to hospital, from whom is the patients sent, when they are sent etc. may disturb the results by the fact that we only had the healthiest patients. Moreover, the ICPC-2 diagnostic system is designed for primary care, and the MAWs accept patients who otherwise would have been hospitalised. To encode diagnoses and symptoms in hospital patients, the standard is to use International Classification of Diseases ICD-10 coding system. Thus, the use of ICPC-2 codes might have underestimated the actual diagnoses that were given at the MAWs.

## Conclusion

Our findings show that there is no such as “the typical MAW patient” or a standardised MAW. Primary care physicians still seem to need the extended diagnostic opportunities in hospital. Moreover, patients transferred to hospital during the stay at MAW are in need of more advanced medical treatment, such as intravenous medication and oxygen therapy. This indicates that the MAW represent an intermediate healthcare level between primary and specialist healthcare. These findings emphasise the necessity of a governmental assessment of structure, equipment and range of services at the MAWs. This also includes a discussion about MAWs’ role in the healthcare system, what the MAWs should do, what kind of patients that can be treated at a MAW and what kind of competence and diagnostics are needed.

### Implications for further research

Large randomised controlled trials (RCTs) could provide more definitive evidence of the effectiveness and clinical outcomes of sending patients to a decentralised alternative to hospitals such as a MAW. We have conducted a multicentre RCT in these five MAWs to compare the effectiveness and clinical outcomes of MAWs versus hospitals, and analyses are ongoing.

## Supplementary Information

**Additional file 1.**

**Additional file 2.**

## Data Availability

Datasets generated and/or analysed during the current study are not publicly available due to local ownership of data, but aggregated data are available from the corresponding author on reasonable request.
