# Supplementary material for: Finding good alternatives to hospitalisation: a data register study in five municipal acute wards in Norway
Source: BMC Health Serv Res. 2022 May 30;22:715. doi: 10.1186/s12913-022-08066-3 (PMC9153207; doi:10.1186/s12913-022-08066-3)
Supplement: Supplementary file 1 — Additional file 1. [file 12913_2022_8066_MOESM1_ESM.docx]

Additional file 1

The variable comorbidity is obtained from variable ”co-diagnosis 1” and co-diagnosis 2” in the registry. Observation with following ICPC2 codes are incorporated into the variable comorbidity.

| A-General and, unspecified | A28, A79 |
| --- | --- |
| B-Blood, blood forming organs and immune mechanism | B72, B73, B74,B81,B82,B99 |
| D- Digestive | D74,D75,D76,D77,D80,D84,D92,D94,D97 |
| K- Cardiovascular | K72,K74, K75, K76 ,K77 ,K78 ,K79 ,K80, K83 ,K85 ,K86, K87,K88,K90,K91,K92,K93;K99 |
| L-Musculoskeletal | L71 |
| N-Neurological | N07,N08,N18,N19,N28,N29,N70,N74,N80,N81,N85,N86,N87,N88 |
| P-Psychological | P05,P15,P16,P18,P19,P20,P70,P71,P72,P73P74,P75,P76,P77,P78,P80,P82,P85,P86,P98 |
| R-Respiratory | R79,R84,R85,R86,R92,R96 |
| T-Endocrine/metabolic and nutritional | T05,T08,T73,T83,T86,T87,T89,T90,T91, T99, |
| U-Urological | U14,U75,U76,U77 |
| X-Female genital | X75,X76,X77 |
| Y-Male genital | Y77 |
