# Supplementary material for: Finding good alternatives to hospitalisation: a data register study in five municipal acute wards in Norway
Source: BMC Health Serv Res. 2022 May 30;22:715. doi: 10.1186/s12913-022-08066-3 (PMC9153207; doi:10.1186/s12913-022-08066-3)
Supplement: Supplementary file 2 — Additional file 2. [file 12913_2022_8066_MOESM2_ESM.docx]

| **Additional file 2** | | | |
| --- | --- | --- | --- |
| **Table 2 Explanation of variable development: treatment** | | | |
| **Variables** | **Valuenames** | **Capture** | **Coding** |
| **Treatment** | Oral medication, Intravenous fluid therapy, Intravenous medication  Mobilization and pain relief, Nebulizer therapy, Others, Oxygen therapy, Observation, Emptying regime/constipation, Bladder catheterization, Wound therapy  Blood transfusion, Nutritional therapy, Physical therapy | From register. Developed to variables by merging treatment opinions | Each value is coded «1» for yes, «0» for otherwise |
